# Health economics aspects of kidney transplantation in Sicily: a benchmark analysis on activity and estimated savings

**DOI:** 10.3389/fpubh.2023.1222069

**Published:** 2023-12-14

**Authors:** Roberto Cacciola, Francesca Leonardis, Lara Gitto, Evaldo Favi, Salvatore Gruttadauria, Marc Clancy, Massimiliano Veroux, Roberta Angelico, Duilio Pagano, Carmelo Mazzeo, Irene Cacciola, Domenico Santoro, Luca Toti, Giuseppe Tisone, Eugenio Cucinotta

**Affiliations:** ^1^Department of Surgical Sciences, Tor Vergata University, Rome, Italy; ^2^Intensive Care Unit, Organ and Tissue Procurement Policlinico Tor Vergata, Rome, Italy; ^3^Dipartimento di Economia, Università Degli Studi di Messina, Messina, Italy; ^4^General Surgery and Kidney Transplantation, Fondazione IRCCS Ca’ Granda Ospedale Maggiore Policlinico, Milan, Italy; ^5^Clinical Sciences and Community Health, Università Degli Studi di Milano, Milan, Italy; ^6^Department for the Treatment and Study of Abdominal Diseases and Abdominal Transplantation, University of Istituto di Ricovero e Cura a Carattere Scientifico – Istituto Mediterraneo per i Trapianti e Terapie ad Alta Specializzazione, University of Pittsburgh Medical Center Italy, Palermo, Italy; ^7^Department of Surgery and Surgical and Medical Specialties, University of Catania, Catania, Italy; ^8^Institute of Cardiovascular and Molecular Sciences, Glasgow University, Glasgow, United Kingdom; ^9^General Surgery Unit, Organ Transplant Unit, University Hospital of Catania, Catania, Italy; ^10^Department of Human Pathology, Emergency Surgery Unit, Università Degli Studi di Messina, Messina, Italy; ^11^Department of Clinical and Experimental Medicine, Medicine and Hepatology Unit, Università Degli Studi di Messina, Messina, Italy; ^12^Department of Clinical and Experimental Medicine, Nephrology and Dialysis Unit, Università Degli Studi di Messina, Messina, Italy

**Keywords:** kidney transplantation, living donation, organ donation, clinical governance, health economics, access to transplantation, kidney transplant waiting list, benchmarking

## Abstract

**Background:**

International and national registries consistently report substantial differences in kidney transplant (KT) activity despite demonstrable clinical and financial benefits. The study aims to estimate the financial resources gained by KT and produce a benchmark analysis that would inform adequate strategies for the growth of the service.

**Methods:**

We analyzed the KT activity in our region between 2017 and 2019. The benchmark analysis was conducted with programs identified from national and international registries. The estimate of financial resources was obtained by applying the kidney transplant coefficient of value; subsequently, we compared the different activity levels and savings generated by the three KT programs.

**Findings:**

The KT activity in the region progressively declined in the study years, producing a parallel reduction of the estimated savings. Such savings were substantially inferior when compared to those generated by benchmark programs (range €18–22 million less).

**Interpretation:**

The factors influencing the reduced KT activity in the study period with the related “foregone savings” are multiple, as well as interdependent. Organ donation, access to the transplant waiting list, and KT from living donors appear to be the most prominent determinants of the observed different levels of activities. International experience suggests that a comprehensive strategy in the form of a “task force” may successfully address the critical areas of the service reversing the observed trend. The financial impact of a progressively reduced KT activity may be as critical as its clinical implications, jeopardizing the actual sustainability of services for patients with end-stage kidney disease.

## Introduction

The clinical benefits of successful kidney transplantation (KT) have been consolidated over the course of the last decades by a wealth of evidence that has been globally reproduced. Similarly, the cost-effectiveness of any type of KT compared to other forms of renal replacement therapy (RRT) has been unequivocally demonstrated (1).

Despite the overwhelming evidence indicating the clinical advantages offered by KT for the treatment of eligible patients with end-stage kidney disease (ESKD), associated with the financial savings produced in favor of any healthcare system, the database of the International Registry of Organ Donation and Transplantation (IRODaT) reports substantial variations of the rate of KT/per million population (pmp) between different countries (2). Noticeably, similar differences may be also observed in regions and territories of countries with developed economies and successful ODT programs (3).

The *Centro Nazionale Trapianti* (CNT), the regulatory body of organ donation and transplantation (ODT) in Italy, has consistently reported substantial differences in the organ donation (OD) and KT activity between Italian regions (4).

The study aims to analyze the KT activity in Sicily and estimate the financial resources obtained; subsequently, we produce a benchmark analysis with national and international programs in comparable regions and territories.

The recently proposed methodology (5) that we used in our study for the financial analysis produces results that may constructively inform the wider transplant community, stakeholders of the KT services, as well as decision-makers on future policies and growth strategies beyond the boundaries of our regional program.

## Methods

Our study is articulated in two components. In the first part, we have analyzed the OD and KT activity in Sicily between 2017 and 2019. To contextualize the performance of the service, as well as define a potential scope for growth in the region, we have identified two demographically comparable ODT programs that we used for the benchmark analysis.

In the second part of the study, we focused on the financial resources generated by KT activity in the study period.

### Rationale for choosing national and international benchmarks

The healthcare system in Italy is devolved to Regional Governments; therefore, the commissioners of the ODT services are part of a healthcare structure that is similar across all 20 Italian regions. In addition, Sicily is an *Autonomous Region* allowing legislation on local matters to be promulgated independently from the National Government.

In Italy, there are no other *Autonomous Regions* with a number of residents similar to Sicily; therefore, we used as a national benchmark for OD and KT activity, the Central Italy region of Lazio that has a number of residents close to Sicily.

The search for a comparable international ODT program to be utilized as a benchmark excluded National programs as comparing a regional with a national healthcare service may be influenced by several biases. Therefore, we searched the IRODaT database for countries with similar population and OD rates to Italy from which we could extract and compare regional data.

The only country reflecting such comparable characteristics with Italy is the United Kingdom (UK) (2). In addition, in the United Kingdom, the devolution of powers to the *Home Nations* (England, Scotland, Wales, and Northern Ireland) on a number of domestic matters including healthcare offers a pertinent similarity with the *Autonomous Region* status of Sicily. In this context, Scotland with a number of residents similar to Sicily and Lazio represents a plausible international benchmark for the benefit of our study.

### Data source

The OD and KT activity data of Sicily were obtained from the database of the Regional Center for Transplantation, *Centro Regionale Trapianti Sicilia* (CRTS) (6), and cross-referenced for accuracy with the official national activity reports produced by the CNT (4). The comprehensive regional database includes the ODT activity, the waiting list for a KT from a deceased donor (KTWL), the prevalence of patients with ESKD, and their modality of RRT.

The data for the benchmark comparison were extracted from the reports of the Regional Center for Transplantation Lazio, *Centro Regionale Trapianti Lazio* (CRTL), Regional Registry of Dialysis and Transplant Lazio (RRDTL), and Scottish Renal Registry (SRR) (7, 8). For accuracy, the data were cross-referenced with the reports published by the relevant national authorities, respectively, CNT and NHS Blood and Transplant (NHSBT) (4, 9, 10).

The Scottish Renal Registry data are reported by calendar year, while NHSBT reports by financial year; therefore, the OD and KT reports for Scotland refer to the period from 1 April 2017 to 31 March 2020.

### Analysis of organ donation and kidney transplantation activity

The comparability of the national ODT programs in Italy and the United Kingdom was obtained through data extracted from the IRODaT database. The OD activity was evaluated through the rate/pmp of Utilized Donors (UD) defined as *“donors from whom at least one organ was transplanted”* (11). The rate of UD was subdivided into Utilized Donors from Donors after Brain Death (UDBD) and Utilized Donors from Donors after Circulatory Death (UDCD). Almost all deceased donors in Sicily and Lazio were Donors after Brain Death (DBD), as in the study period the Donation after Circulatory Death (DCD) program was at the initial implementation stage in Sicily, while in Lazio had not yet started.

The national rate of KT/pmp was also included in our analysis, and it was divided into kidney transplant from living donor (KTLD) and kidney transplant from deceased donor (KTDD). The specific typology of KT in Sicily was compared with the respective number of kidney transplants from donors after brain death (KTDBD), KT from DCD, and KTLD in the benchmark programs.

We analyzed the OD and KT activity in Sicily, Lazio, and Scotland for 3 consecutive years, from 1 January 2017 to 31 December 2019. The years 2020 and 2021 have not been included because the ongoing SARS-CoV-2 pandemic did substantially influence ODT activity nationally and internationally (12).

The selected benchmark OD and KT activity programs of Lazio and Scotland will be referred, respectively, as National Benchmark 1 (NBench1) and International Benchmark 2 (IBench2).

#### Kidney transplantation vs. dialysis

The landmark study of Wolfe et al. (13) demonstrating the survival advantage of patients receiving KT from a deceased donor (DD) vs. RRT paved the way to a wealth of evidence that has been globally reproduced over the subsequent years.

The continuous expansion of KT practice has confirmed not only the survival advantages but also established the superiority in terms of quality-adjusted life years (QALY) of KT vs. any other form of RRT (1), also when considering more challenging and more diverse typology of donors, such as living donors (LD) with blood group incompatible, extended criteria donors (ECD), or donors after circulatory death (DCD).

The survival and QALY advantages of KT are also associated with demonstrable savings. Such savings translate into financial benefits for the healthcare services, naturally extending into wider benefits for the society matured through the return to normal or near normal personal and working life of patients with ESKD. Remarkably, such savings have been quantified allowing a realistic calculation of the financial benefits produced by KT. Among the several publications confirming the positive financial impact of KT, we have identified two separate reports produced by the regulatory bodies of organ donation and transplantation in Italy and in the United Kingdom (CNT and NHSBT). Both national authorities achieve almost identical conclusions regarding the costs of RRT and KT, as well as both indicate that the savings initiate after the first year of a successful KT; thereafter, the savings calculated from the second year of a successful KT are substantially similar. For the benefit of our study, we used the demonstrated saving of €25.000 per year per functioning single KT from the second year of transplantation (14, 15).

#### Metrics used for the financial analysis

The metrics that we used for the financial analysis are reproduced from a recently published study merging the fixed financial parameters (tariff and savings) together with the reported efficacy of treatment (rate of functioning and non-functioning KT) that are subsequently applied to the efficiency of the service (actual number of KT performed per year).

The reimbursement costs represented by the tariff paid by regional commissioners to healthcare providers (HP) together with the estimated savings produced by each functioning KT, including the tariff costs of each non-functioning KT, represent the financial parameters.

KT Tariff in € = 33.162

Savings in € per functioning kidney transplant per year after first year of KT = €25.000

The efficiency and efficacy parameters used are represented by the total number of KT performed together with the estimated rate of functioning and non-functioning KT extracted from the governance reports of the national regulatory bodies.

Estimated functioning kidney transplant (eFKT) = 80% of total number of KT

Estimated non-functioning kidney transplant (eNFKT) = 20% of total number of KT

These metrics allow a realistic estimate of the savings produced by KT. Specifically, the estimated gross savings (eGrSavs) are achieved by subtracting from the demonstrable savings (obtained by eFKT) the costs inflicted by eNFKT according to the formula:

(eFKT × 25.000) - (eNFKT × 33.162) = eGrSav

The laborious calculation may be simplified by the use of a coefficient defined as the kidney transplant coefficient of value (KTCoV). Such coefficient is produced using the same parameters used to calculate the eGrSav and dividing the result by the actual number of KT performed according to the formula:

(eFKT × 25.000) - (eNFKT × 33.162) / Total number of KT = KTCoV

Or

eGrSav/n. KT = KTCoV

The KTCoV being the product of fixed financial parameters (tariff and savings) and variables (eFKT and eNFKT) that are obtained from the actual denominator (total number of KT) is constant regardless of the number of KT retrospectively or prospectively for any year of KT activity analyzed.

The value that we obtained for the KTCoV is €13.367,6.

The simulations reported in the supplement, with the hypothetical number of 50, 100, or 150 KT per year, confirm that the KTCoV is a constant value as already reported (5); therefore, it will be used in our study for the financial analysis and estimates.

#### Financial analysis

The savings produced by KT initiated after 1 year of successful KT; therefore, our calculations are based on the activity observed in Sicily and in the benchmarks program in the years 2017–2019 and the savings estimated for the following years, starting from 2018.

The calculation of the estimated gross saving (eGrSav) was achieved by applying the KTCoV to the number of KT. Available evidence applicable to our study indicates that the KTCoV consists of €13.367,6 (5). The analysis aims to

1. calculate the eGrSav produced by the actual KT activity in Sicily in the study years (2017–2019) and
2. compare the eGrSav of Sicily with the Benchmark KT programs for the years 2020, 2021, and 2022.

### Statistical analysis

This study is not designed to evaluate a statistically significant difference in the efficiency between the KT program in Sicily and the benchmark programs that we have selected. However, we focused our attention on the different typologies of KT as determinants of the overall activity; therefore, we calculated, where appropriate, with Fisher’s exact test whether the typology of KT may show a statistically significant difference.

The savings produced by KT have not been compared with a statistical methodology as even non-significant differences in finances may still be highly relevant for the commissioning authorities, depending on the status of healthcare service and overall expenditures.

## Results

### National context and comparability

The population of Italy and the United Kingdom is reported as consisting of 60.4 million and 68.5 million, respectively (16). The rate of UD confirming the comparability of both national OD activities is reported in Table 1. Noticeably, in Italy, the rate/pmp of UDBD is constantly higher than in the United Kingdom, while the KT/pmp rate is substantially higher in the United Kingdom.

**Table 1:** Utilized donors and kidney transplants in Italy and the UK (2).

	2017		2018		2019	
	Italy	United Kingdom	Italy	United Kingdom	Italy	United Kingdom
UD/pmp (Cumulative)	27.7	21.34	22.6	23.35	22.8	23.01
UDBD/pmp	27.2	13.18	21.82	14.79	21.7	13.86
UDCD/pmp	0.5	8.16	0.78	8.56	1.1	9.15
KT/pmp (Cumulative)	41.3	52.95	35.14	55.14	35.3	54.9
KTDD/pmp	36.3	37.84	30.2	39.5	29.7	39.56
KTLD/pmp	5	15.11	4.94	15.64	5.6	15.34

### Results of organ donation and kidney transplantation activity

The regional and national registries demonstrate consistency of data. The CRTS reports indicate that 71 Sicilian patients received a KT out of the region in the study period. These patients were excluded from our analysis, although such relevant cohort deserves separate considerations. The report of the RRDTL does not include information on the prevalent ESKD patient resident in the region receiving RRT in the form of a KT. However, the absence of such specific information does not affect the focus of our study.

The rate of UD in Sicily declined from 15.4/pmp in 2017 to 8/pmp in 2019. In addition, the overall number of KT progressively decreased from 160 in 2017 to 101 in 2019.

The detailed typology of UD and KT divided by year and in comparison with NBench1 and IBench2 is reported in Table 2.

**Table 2:** Yearly comparison of RRT population, UD, and KT.

	Sicily	NBench1	IBench2
2017	//	//	//
RRT prevalence	5,106	*	5,177
- HD	4,328 (84.7%)	4,340	1959 (38%)
- PD	235 (4.6%)	356	194 (4%)
- KT	543 (10.7%)	NA	3,024 (58%)
Waiting list	529	922	402
UD/pmp (DBD/DCD)	15.4	24.4	19.3 (11.3/8.0)
KT (Total number)	160	262	287
- KTDD (DBD/DCD)	151	236	203 (128/75)
- KTLD	9	26	84
2018	//	//	//
RRT prevalence	5,056	*	5,318
- HD	4,199 (83%)	4,398	1942 (37%)
- PD	224 (4.5%)	334	215 (4%)
- KT	633 (12.5%)	NA	3,161 (59%)
Waiting list	568	910	439
UD/pmp (DBD/DCD)	9	24.4	17.9 (12.5/5.4)
KT (Total Number)	118	263	263
- KTDD (DBD/DCD)	109	222	167 (113/54)
- KTLD	9	41	96
2019	//	//	//
RRT prevalence	5,126	*	5,436
- HD	4,183 (81.6%)	4,534	1919 (35%)
- PD	221 (4.3%)	348	213 (4%)
- KT	722 (14.1%)	NA	3,304 (61%)
Waiting list	551	958	409
UD/pmp (DBD/DCD)	8.2	21	18.4 (11.9/6.5)
KT (Total number)	101	223	280
- KTDD (DBD/DCD)	90	181	182 (113/69)
- KTLD	11	42	98

*RRDTL does not report the exact number of patients receiving RRT with a KT in the region (7).

The average number of patients on hemodialysis (HD) observed in Sicily is similar to the NBench1 (4,327 vs. 4,424) but substantially superior to IBench2 (4,327 vs. 1940). However, the average number of patients on the KTWL in Sicily is substantially inferior to NBench1 (550 vs. 930) and superior to IBench2 (550 vs. 417). Specifically, the KTWL in Sicily represents 12% (550/4554) of the HD and Peritoneal Dialysis (PD) population combined, while in NBench1 and IBench2 are both 19.5% (930/4770 and 417/2147).

There are in total 379 KT reported in Sicily in the study period, while the NBench1 and IBench2 reports show a substantially superior number, respectively, 748 and 830. Notably, the number of KT from DBD in Sicily is similar to IBench2, with a calculated rate/pmp actually superior (23.6 vs. 21.4). The number of KTLD in Sicily is substantially inferior to both the NBench1 and IBench2 (Table 3) with a calculated annual rate of 1.9/pmp in Sicily, 6.6/pmp for NBench1, and 16.8/pmp for IBench2. The typology of KT practice with a higher number of KTLD in both NBench1 and IBench2 programs compared to Sicily is also statistically significant, as summarized in Table 4. In addition, the substantial number of KT from DCD in IBench2 is not comparable with the activity in Sicily representing the initial experience.

**Table 3:** Cumulative comparison of RRT population, UD, and KT.

	Sicily actual	NBench1	IBench2
Average number of prevalent patients with ESRD by type of RRT 2017–2019	5,197	*	5,310
HD	4,327	4,424	1940
PD	227	346	207
KT	643	NA	3,163
Average number of patients on KT Waiting List 2017–2019	550	930	417
Average UD/pmp (DBD/DCD) 2017–2019	10.8 (10.8/0)	23.2 (23.2/0)	18.5 (11.9/6.6)
Cumulative number of KT 2017–2019	379	748	830
KT DBD	348 (91.8%)	639 (85.4%)	354 (42.7%)
KT DCD	2 (0.6%)	0	198 (23.8%)
KTLD	29 (7.6%)	109 (14.6%)	278 (33.5%)

*RRDTL does not report the exact number of patients receiving RRT with a KT in the region (7).

**Table 4:** Comparison of activity by different types of KT.

	KTDD	KTLD	*p*-value
Sicily	350	29	//
NBench1	639	109	0.0007
IBennch2	552	278	0.0001

### Results of financial analysis

The savings produced by KT mature after the first year of transplantation. The eGrSav is calculated by applying the KTCoV (€13.367,6) to the yearly KT activity; therefore, it is directly proportionate to the number of any type of KT performed.

1. The calculated eGrSav is the product of the previous year KT activity; therefore, in 2018, only the activity observed in 2017 may be taken into consideration. Consequently, in 2019, the eGrSav is calculated from the KT activity in 2017 and 2018. Finally, the KT activity of the years 2017, 2018, and 2019 produces the eGrSav of matured in 2020 (Table 5).
2. The same eGrSav matured in 2020 is also available for the years 2021 and 2022. The savings produced by the limited activity during the pandemic years 2020 and 2021 are not included in the study.
3. In Sicily, the estimated savings between 2018 and 2022 reached the amount of €21.053.970. The detailed year-by-year eGrSav is reported in Table 5.
4. The comparison between Sicily and benchmark programs reveals that the cumulative eGrSav matured in the study period was substantially less than both NBench1 and IBench2 (€21.053.970 vs. €40.517.195,6 and €44.474.005,2). The detailed year-by-year eGrSav reported in Table 5 demonstrates the savings accrued in the study period.
5. In Sicily, the highest savings were produced in 2018 (€2,138,816) from the activity of 2017 (160 KT), while the lowest savings were €1.350.127,6, those were calculated from the activity reported in 2019 (101 KT) that represented 26.6% of the savings produced in 2020 (€5.066.320,4). The eGrSav produced by the NBench1 and IBench2 is similarly consistent over the years of the study period, as well as being both considerably superior to those generated by the Sicilian program.

**Table 5:** Comparison of yearly and 5-year cumulative estimated savings in €.

	Sicily	NBench1	IBench2
2017*	//	//	//
eGrSav in 2018	2.138.816	3.502.311,2	3.836.501,2
eGrSav in 2019	3.716.192,8	7.017.990	7.352.180
eGrSav in 2020	5.066.320,4	9.998.964,8	11.095.108
eGrSav in 2021**	5.066.320,4	9.998.964,8	11.095.108
eGrSav in 2022**	5.066.320,4	9.998.964,8	11.095.108
Cumulative 5-year eGrSav (2018–2022) from KT activity 2017–2019	21.053.970	40.517.195,6	44.474.005,2

*No Savings in 2017 activity as savings begin 1 year after KT. **Cumulative Savings produced only by KT activity in the study period (2017–2019).

## Discussion

The acknowledgement that “health financing is a core function of health systems” (17) amplifies the importance of the financial savings that may be produced through improved clinical performance. As importantly, benchmarking the activity volumes and processes contributes to care quality improvement (18). However, finding the most appropriate realistically achievable benchmarks and indicators may be highly challenging, particularly so, in ODT where numerous variables may substantially affect the performance of a program.

Our efforts in finding suitable benchmarks for Sicily were inspired by identifying realistic terms of comparison that could generate achievable objectives, rather than performing an ineffectual comparison with historically highly performing ODT programs *per se* such as those in Spain, the US, or in countries with a smaller population. In this spirit, we opted to analyze exclusively categorical data omitting the production of a statistical analysis of the savings, despite the data we report in our study may be amenable to produce significant *p*-values, when comparing KT activity and savings between Sicily and ODT benchmarks programs.

The considerations on the KT activity and its financial implications in Sicily necessarily require an adequate contextualization with the OD performance and volume of the KTWL.

The observed sharp decline of 47% of UD/pmp (from 15.4/pmp in 2017 to 8.2./pmp in 2019) in Sicily has inevitably impacted on the number of organs available for transplantation in the region and nationally. Available evidence suggests that in Italy, each UD generates on average three organs available for transplantation. Accordingly, five UD may lead to eight kidney transplants (4, 5). The reduction of UD consisting in 6.4/pmp and 7.2/pmp, respectively, in 2018 and 2019 reported in Sicily would account for 66 less UD; consequently, it would translate in at least 104 less kidneys available for the patients active on the KTWL.

Several factors may influence the UD/pmp rate; in particular, it would be valuable establishing whether a progressive contraction of the number of P*otential Donors* (11) is associated with a reduction of consent to donation. Undoubtedly, the combination of such events would lead to a reduced number of UD. Although a regional strategy integrated with the national OD framework will address a consent rate of 50% in 2019 (4), the reduced identification of potential donors, if confirmed, may indicate that a review of the processes, as well as of the service infrastructures and workforce, should be considered.

While the number of organs available for transplantation is the product of the efforts produced by the regional OD network, culminating in the UD rate, another pivotal factor affecting the KT activity is represented by the number of patients active on KTWL.

In Sicily, the proportion of patients with ESKD receiving RRT in the form of HD/PD who are active on the KTWL is inferior to both NBench1 and IBench2. The analysis of the reported data shows that the average number of patients active on the KTWL in Sicily and NBench1 is substantially different (550 vs. 930). Although such observation may suggest that access to transplantation may be highly efficient in NBench1, it is noted that during the study period, an average of 38.5% of KT per year is performed on non-residents of the region (7). In this regard, it should be highlighted that in Italy, patients eligible for a KT may be activated on two regional KTWLs on their request. Implicitly, the evaluation of the regional pathways supporting ESKD patients toward access to transplantation in NBench1 may be arduous if based on this single observation; furthermore, such specific comparison with Sicily may be biased by the presence of more transplant centers (TxC) in Nbench1; five TxC in NBench1 vs. three in Sicily. The discrepancy of the KTWL population between Sicily and NBench1 may therefore be explained by the fact that a substantial number of patients active on the regional KTWL of NBench1 are actually residents of other Italian regions. In support of this explanation, we notice that despite a higher KT activity in NBench1, the number of patients on HD/PD remains very similar to Sicily. A further potentially critical similarity between Sicily and NBench1 is represented by the fact that in both regions, almost 70% of the HD centers are private (6, 7).

The average HD/PD population in IBench2 is substantially inferior to Sicily: 53% less (2,147 vs. 4,554). In addition, the KTWL in IBench2, despite consisting of an inferior number of patients (417), it still produces a higher rate of patients waiting for a KT than Sicily (19.5% vs. 12.5%).

Notwithstanding the fact that it may be arduous finding a valid explanation for the limited access to transplantation, as also reported by other developed ODT programs (19, 20), it may be relevant highlighting that the HD/PD services in IBench2 are entirely public and provided by the National Health Service, while in Sicily such service is largely delivered by private healthcare providers (6). This observation indicates that adequate governance measures should be implemented to ensure that such prominence of private HD providers might not affect the access to the KTWL, hence limiting the option of KT that remains the gold standard of treatment for patients requiring RRT (21).

In the light of these observations, it may be sustained that the pool of patients who would be eligible for a KT in Sicily is currently underrepresented by the regional KTWL. Therefore, access to transplantation represents a critical aspect of the regional ODT services. In particular, adherence to the *Kidney Disease: Improving Global Outcome* (KDIGO) guidelines (22), concerning access to transplantation, would require the enhancement and consolidation of the interactions between the renal network and transplant centers (TxC).

The overall number of 379 patients who had a KT in Sicily produces a yearly average rate of 25.3/pmp in the study period, which is substantially below the national Italian average of 37/pmp.

The cohort of 71 patients, who were transplanted out of the region in the study period, is a further contributing factor in generating a wider gap between regional and national average of KT/pmp. In fact such difference would be reduced if the KT would have been carried out in one of the Sicilian TxC. The expected costs for this cohort, generated by a tariff of €33.162 per KT, amount to €2.354.502. However, such expected costs may be an underestimate as out of the region healthcare providers (HP) may apply different tariffs. In addition, patients may be entitled to the reimbursement of part of the travel and subsistence expenses representing a further aspect of the social costs of the phenomenon of “internal emigration” for KT that is met primarily by patients.

The difference between the calculated eGrSav of Sicily with those generated by the KT activity in NBench1 and IBench2 may be regarded as substantial *“Foregone Savings”* for the Sicilian healthcare services, strengthening, beyond the established clinical benefits, the scope for growth of the KT service (Table 5).

The contributing factors may be multiple, and some may be corrected or mitigated. The diversification of KT typology observed in IBench2 that includes high rates of KT from DCD and KTLD allows the service not to rely exclusively on DBD. The data analyzed, in fact, indicate that the rate of UDBD and average number of KT from DBD in Sicily and IBench2 are substantially identical. Consequently, the reduction of the KTWL in IBench2 reflects the fact that the majority of patients with ESRD (60%) receive RRT in the form of a KT, because of the more diversified typology of KT including DCD and KTLD.

Such consolidated practice that has been sustained in the course of the last decade in IBench2 has undoubtedly contributed to achieve substantial savings for the wider healthcare service. It may be argued that if the same KT activity was replicated in Sicily over the study period, the savings over the 3 following years may exceed €50 million as suggested by our estimate. Noticeably, the eGrSav produced in our analysis (Table 5) represents the minimal savings that can be obtained from KT activity. In fact, the reports of both CNT and NHSBT indicate a sustained GS at 5 years well above 80%; hence, indicating that the actual savings produced by a superior number of functioning kidney transplants could be more conspicuous than actually indicated in our analysis.

The benchmark activity identified for the benefit of the study may be reproducible in Sicily, provided that a comprehensive strategy recognizing ODT as a unique healthcare entity scientifically and financially interdependent may be designed by the stakeholders of the service (5). The successful implementation of adequate measures aimed to reverse the observed trend in OD and KT could be addressed comprehensively with the institution of a regional “task force” integrated with a national strategic plan of the growth of the service.

International experience in this regard supports such approach. The most clear example of a successful implementation of a comprehensive strategy may be identified in the United Kingdom; where following the institution of the OD Task Force in 2006 and the implementation of its recommendations, it was observed a remarkable countrywide increase of UD and KT in the course of the following decade (23).

A potentially successful pathway that may be followed is represented by the Spoke-and-Hub model aimed to consolidate the ODT network on the island of Sicily. Such model, already suggested and successfully implemented in other healthcare services (24) including Sicily itself (25), may address the critical aspects of the ODT services that we have presented. Undoubtedly, an effective and capillary network strongly linked with the centers of excellence operating in the Sicilian territory would allow more patients to access the better option of RRT represented by KT either from a DD or a LD, ultimately reproducing the well-recognized benefits to the patients, their families, and the finances of the regional healthcare services.

The financial impact of a progressively reducing KT activity may be as critical as the clinical implications of a large population on HD/PD, in particular taking into consideration those patients eligible for a KT who may be suspended from the KTWL (26). The incremental costs of healthcare in the context of a global crisis and financial insecurity, inevitably will jeopardize the sustainability of a number of services. It may be conceived that a progressive investment in resources, parallel to the increment of savings produced, may constitute a realistic budget aimed to guarantee the growth and, ideally, the financial self-sufficiency of the OD and KT services.

Healthcare systems funded by taxpayers may not afford to miss potential financial resources; certainly, regular evaluation of performance following extensive benchmarking processes, as well as constructive clinical governance, would be of paramount importance (27, 28).

The health economics of KT constitutes a highly challenging area of healthcare with undervalued potentials and unexplored benefits. Our study does not have the ambition to address all the issues generated by the health economics of KT; similarly, we do acknowledge the limitations of our study and the proposed model.

The recent proposal of new metrics merging demonstrable financial and clinical outcomes (savings/rate of FKT) requires a more contemporary validation. In fact, the actual costs of RRT, including KT, on which we, as most of the other authors on the topic, have based the financial analysis are now a decade old, or actually older. Furthermore, the continuously evolving practice of organ donation associated with the growing use of new and expensive technologies such as Normothermic Regional Perfusion or *ex situ* organ reconditioning (30) will require to be factored in the general costs of the ODT services affecting also the cost analysis of KT. Relevantly, the necessary expansion of the donor pool, through the implementation of DCD programs, as well as an increased utilization of extended criteria donors aimed to treat an increasingly more complex pool of patients with ESKD, is linked to heterogenous graft survival rates. Although such diversified donor and recipient pool characterizing contemporary practice in KT still produces a survival advantage of KT vs. any other forms of RRT (1) as demonstrated by the governance reports on which we based the rate of FKT for the production of the KTCoV, a more granular and visible governance processes would be required to satisfy the expectations of commissioners, wider transplant community, and patients.

The more accurate definition of a budget generated by the eGrSav produced by KT activity certainly would be gladly received; however, it may generate some repercussions on the management of the allocation of healthcare resources that, in current times, have been progressively restricting. From this perspective, it should be highlighted that our proposed model does not identify a “new” stream of funding; instead, it allows a reliable estimate of financial resources that are already available but not as visible as they could, particularly so in a regional healthcare budget. In our opinion, the funding method for the whole ODT services may benefit from accurate budgeting provided that it will be addressed as a unique and interdependent healthcare entity (5).

Our analysis focusing on the health economics of KT does not include the social costs and financial benefits that the regional community may undoubtedly enjoy. The overall considerations on such beneficial aspects of successful KT would require an accurate evaluation of the return to usual activities not only of the patients with ESKD but also of the positive impact that may be observed on the immediate families.

The methodology we suggest may potentially be applied to any KT program with adequate corrections to the parameters determining the KTCoV. The tariffs paid by commissioners to HP for KT services may vary between regions and countries; similarly, the actual HD/PD cost may be different producing different savings. Therefore, despite the fact that the same method may be applied, it may produce different KTCoV that consequently may be lower or actually higher than the one we have calculated. As importantly, in the same healthcare service that we have analyzed in our study, the actual tariffs may be revised by the commissioners, as well as HD/PD costs may be affected by discount rates and inflation. Necessarily, the application of the methodology we suggest for the calculation of a reliable eGrSav will require regular financial ad clinical outcome auditing to obtain a well-grounded calculation. Although a necessary regular validation of costs and clinical outcomes may be interpreted as a limitation, financial planning offices will easily source the relevant information from HP and regulatory bodies, guaranteeing a solid and reliable calculation of the KTCoV and eGrSav.

In conclusion, our study identifies that in Sicily, a number of critical areas would require the implementation of simultaneous interventions to reverse the current trend of performance. The application of recently proposed health economics metrics applied to the activity of the KT program in Sicily, followed by a benchmark analysis with other programs active in comparable territories, indicates that a progressively improved efficiency of the KT activity could be associated with increased savings that may subsequently lead to potential reinvestments in the ODT services, wider healthcare services, and new technologies aimed to reduce the chronic shortage of organs available for SOT (29).

We trust that our analysis may contribute to advance future dialogs between stakeholders of the services as the timing for the implementation of adequate strategies appears already critical because of the constant reduction of KT activity that was observed in the years preceding the SARS-CoV-2 pandemic; necessarily, it requires now the highest level of attention.

## Data availability statement

The original contributions presented in the study are included in the article/supplementary material, further inquiries can be directed to the corresponding author.

## Author contributions

RC, FL, LG, EC, IC, LT, DS, and GT: rationale of the study and original draft of the manuscript. RC, EF, LT, IC, and RC: final revision. SG, DP, CM, MV, MC, and IC: data collection and interpretation. CM and RA: literature review. LG, FL, and EF: editing. RC, LT, and EC: supervision. LG, IC, and RA: logistics. All authors contributed to the article and approved the submitted version.
